# Identification of the Novel *Nup188-brr7* Allele in a Screen for Cold-Sensitive mRNA Export Mutants in *Saccharomyces cerevisiae*

**DOI:** 10.1534/g3.118.200447

**Published:** 2018-07-18

**Authors:** Anne de Bruyn Kops, Christine Guthrie

**Affiliations:** Department of Biochemistry and Biophysics, UCSF, San Francisco, CA 94143

**Keywords:** mRNA export, *NUP188*, *brr7*, *GLE1*, *brr3*

## Abstract

The maturation and export of mRNA from the nucleus through the nuclear pore complex is critical for maintaining an appropriate proteome in all eukaryotic cells. Here we summarize a previously unpublished screen in S. cerevisiae that utilized an established dT50 *in situ* hybridization assay to identify cold-sensitive mutants that accumulated bulk poly A RNA in the nucleus. The screen identified seven mutants in six complementation groups, including the *brr6-1* strain that we described previously. In addition to *brr6-1*, we identified novel alleles of the key transport gene *GLE1* and *NUP188*, a component of the Nic96 nucleoporin complex. Notably, we show that the *nup188-brr7* allele causes defects in select protein import pathways as well as mRNA export. Given recent structural and functional evidence linking the Nic96 complex to transport components, this mutant may be particularly useful to the transport community.

Gene expression in eukaryotes requires export of messenger RNAs (mRNAs) from the nucleus prior to their translation in the cytoplasm. Many factors involved in this process have been identified, leading to a clear conceptual framework for export wherein mRNAs undergo co-transcriptional processing and association with numerous factors that mediate their stability, association with the nuclear pore complex (NPC), release into the cytoplasm, and ultimately their translational fate (reviewed, Okamura *et al.* 2015; Knockenhauer and Schwartz 2016; Williams *et al.* 2018). However, the mechanisms by which these events are coordinated are not well understood. In addition, recent evidence for selective and cell-type-specific mRNA export in higher eukaryotes, along with connections between export and human disease, emphasizes the need to understand how export is regulated.

The study of mRNA export mutants in budding yeast has contributed enormously to our current knowledge of export (reviewed, Floch *et al.* 2014) and continues to be a powerful tool in understanding how mRNAs interact with the NPC during export. Two *in situ* hybridization screens aimed at identifying temperature-sensitive (ts) mutants that accumulate mRNA in the nucleus (Amberg *et al.* 1992; Kadowaki *et al.* 1992) were successful in identifying various mRNA maturation and export-related components. However, only about 30% of essential genes give rise to detectable ts mutants following *in vivo* mutagenesis in Saccharomyces cerevisiae (Diehl and Pringle 1991; Harris and Pringle 1991), suggesting that additional export components might be identified by screening other types of banks.

Different genes have been identified via cold-sensitive (cs) *vs.* ts phenotypes in bacteriophage (Scotti 1968; Cox and Strack 1971; Jarvik and Botstein 1975) and yeast (Moir *et al.* 1982) and we were previously successful in identifying novel spliceosome components not found in ts screens (Noble and Guthrie 1996) using a bank of 350 cold-sensitive yeast strains (Moir *et al.* 1982). Therefore, we screened the same cs bank by *in situ* hybridization screen in an effort to identify novel export factors or novel alleles that may reveal additional functions of known factors.

The screen yielded seven cs transport mutants in six complementation groups (*brr3*, *brr4*, *brr6*, *brr7*, *brr8* and *brr9*). We cloned genes for the *brr3*, *brr6* and *brr7* mutants: *BRR6* encodes a nuclear envelope integral membrane protein, Brr6, (de Bruyn Kops and Guthrie 2001) required for normal envelope lipid homeostasis, nuclear pore complex (NPC) and spindle pole body assembly (reviewed, Schneiter and Cole 2010; Jaspersen and Ghosh 2012) and transcriptional regulation (A. de Bruyn Kops, J. E. Burke, C. Guthrie, unpublished data). The mutants *brr3-1* and *brr7-1*, are novel alleles of *GLE1* (Murphy *et al.* 1996) and *NUP188* (Nehrbass *et al.* 1996; Zabel *et al.* 1996) respectively. Gle1 is a key NPC-associated protein involved in release of mRNAs into the cytoplasm (reviewed, Folkmann *et al.* 2014). Nup188 is a component of the Nic96 complex that forms the central scaffold of the NPC (reviewed, Vollmer and Antonin 2014). We provide preliminary characterization of the *nup188-brr7* allele that demonstrates effects on RNA export and selective protein import pathways not seen in other *nup188* mutants. Recent structural and functional analyses have suggested previously unknown relationships between the Nic96 complex and nuclear transport that are being explored by several labs. Therefore, we anticipate that the *nup188-brr7* mutant could prove useful in dissecting the connection between the Nic96 complex and nuclear transport.

## Materials and Methods

### Strains and Plasmids

The yeast strains used in this study are listed in Table 1. The original *NUP188* clone was isolated from the Rose genomic library (Rose *et al.*, 1987). Other Nup188 plasmids were constructed as described below. Other constructs used in this study were pGFP-URA (SV40 NLS-GFP), pRS315-NPL3-GFP++ (Npl3-GFP), pUN100-PNOP1-GFP-MTR10 (Mtr10-GFP), pL25-(GFP)3 (L25-GFP), and pRS316-pHIS-NAB2-GFP (Nab2-GFP, derived from pRSpHIS-NAB2-GFP).

**Table 1 S.** *cerevisiae* strains used in this study

Name	Relevant genotype	Background genotype	Source
DBY4157-4482	Cold-sensitive Mutants	Derived from mutagenized DBY473 (see below)	D. Botstein
DBY4483-4497	Cold-sensitive Mutants	Derived from mutagenized DBY640 (see below)	D. Botstein (Moir *et al.*, 1982)
DBY473	Wild-type strain	(S288C background) *Mat* ***α*** *gal- mal- his4-619*	D. Botstein (Moir *et al.*, 1982)
DBY640	Wild-type strain	(S288C background) *Mat* ***a*** *gal- mal- ade2*	D. Botstein (Moir *et al.*, 1982)
YPH399	Wild-type strain	(S288C background) *Mat* ***α*** *his3-∆200 leu2-∆1 ura3-52 trp1-∆63 ade2-101 lys2-801*	P. Hieter
*kap104-16*	*kap104* –ts plasmid covering *kap104* deletion	(DF5 background) *kap104∆*::*ura3*::*HIS3*, *kap104-16-TRP1*	J. Aitchison (Aitchison *et al.* 1996)
*KAP104*-WT	*KAP104*-Wild type plasmid covering *kap104* deletion	(DF5 background) *kap104∆*::*ura3*::*HIS3*, *KAP104-URA3*	J. Aitchison (Aitchison *et al.* 1996)
yDBK1(*brr3-1*)	*brr3*	(YPH399 outcross of DBY4217) *MAT****α****his3-∆200 lys2-801 leu2-∆1 ura3-52*	This study and Noble and Guthrie, 1996
yDBK2*(brr4-1)*	*brr4*	(YPH399 outcross of DBY4475) *MAT****α*** *ade2-101 trp∆63 lys2-801 ura3-52*	This study and Noble and Guthrie, 1996
yDBK3*(brr6-1)*	*brr6*	(YPH399 outcross of DBY4275) *MAT****α*** *his3-∆200 lys2-801*	This study
yDBK4*(brr6-2)*	*brr6*	(YPH399 outcross of DBY4412) *MAT****α*** *his-∆200 lys2-801*	This study
yDBK5*(brr7-1)*	*nup188-brr7*	(YPH399 outcross of DBY4345) *MAT****α*** *ade2-101 trp∆63 lys2-801 ura3-52*	This study
yDBK6*(brr8-1)*	*brr8*	(YPH399 outcross of DBY4383) *MAT****α*** *his3-∆200 ura3-52 ade2-101*	This study and S. Noble and C. Guthrie, unpublished data
yDBK7*(brr9-1)*	*brr8*	(YPH399 outcross of DBY4467) *MAT****α*** *ade2-101 trp∆63 lys2-801 leu-∆1 ura3-52*	This study
yDBK17	*nup188-brr7*with *URA3*-marked *NUP188* integrated at *NUP188 l*ocus	(from yDBK5(*brr7-1*), *NUP188-URA3*	This study
*nup188∆*::*HIS3*	*nup188∆*::*HIS3* knockout in W303	*ade2-1 ura3-1 his3-11*, *15 trp1-1 leu2-3,112 can 1-100 nup188∆*::*HIS3*	R. Wozniak (Nehrbass et al., 1996)
yDBK15	*nup188∆*::*LEU2* knockout in YPH399	*Mat* ***a*** *his3-∆200 leu2-∆1 ura3-52 trp1-∆63 ade2-101 lys2-801 nup188∆*::*LEU2*	This study
yDBK16	*nup188∆*::*LEU2* knockout in YPH399	*Mat* ***α*** *his3-∆200 leu2-∆1 ura3-52 trp1-∆63 ade2-101 lys2-801 nup188∆*::*LEU2*	This study
*∆nup188 kap104-ts*	*nup188∆*::*LEU2,kap104-ts*	(from *nup188∆*::*LEU2* x *kap104-16*) *nup188∆*::*LEU2 kap104∆*::*ura3*::*HIS3*, *Kap104-ts(TRP1)*	This study
PSY1042	*∆kap123*, *pse1-1*	*Mat* ***a*** *his3∆100 ura3-52 leu2∆1 trp1∆63 kap123∆*::*HIS3 pse1-1*	P. Silver (Seedorf and Silver, 1997)
MS4 1	WT strain isogenic with PSY1042	*Mat* ***a*** *ura3-52 leu2∆1 trp1∆63*	P. Silver (Seedorf and Silver, 1997)

### Screen for cold-sensitive export mutants

A previously characterized bank of 350 cold-sensitive mutants of S. cerevisiae (Moir *et al.* 1982) was screened using a dT50 *in situ* hybridization assay (see below) in batches of approximately 50 mutants along with wild type control. Cells were grown at 30° and shifted O/N to16° before fixation at OD600≅0.3. Strains showing nuclear accumulation of mRNA were retested in the same manner to confirm the phenotype.

Candidate cs mRNA export mutants were crossed 2- 4 times to wild type strains derived from S288C (either DBY640 or DBY473 for the first cross and then YPH399 in subsequent matings). The resulting diploids (heterozygous for the cs mutation) were sporulated. All showed 2:2 segregation of the cs phenotype following the final cross. Linkage between cs growth and mRNA export defects was established by examining the progeny of 5 tetrads for both growth and mRNA export defects at 16°. mRNA export defects were assessed by *in situ* hybridization following a 10 hr cold-shift. Complementation analysis was carried out by mating a matrix of a and α spores from each mutant at 30° for one day followed by replica plating to pre-chilled YEPD plates and incubating at 16° for 5 days along with wild-type controls. The outcrossed descendents of DBY4217, DBY4345, DBY383, DBY4467 and DBY4475 formed single complementation groups (the mutants were designated *brr3-1*, *brr7-1*, *brr8-1*, *brr9-1* and *brr4-1*) respectively. The descendents of DBY4275 and DBY4412 were allelic (designated *brr6-1* and *brr6-2*) as shown by a 4:0 segregation of the cs phenotype in 10 4-spore tetrads obtained by sporulating the a DBY4275/DBY4412 heterozygous diploid. The *brr6-1* and *brr6-2* strains were subsequently shown to carry the same mutation (de Bruyn Kops and Guthrie 2001).

### In situ hybridization assay

*In situ* hybridization was performed on fixed yeast cells using a protocol similar to those described previously (Amberg *et al.* 1992; Kadowaki *et al.* 1992) Cells were grown in YEPD media and harvested in early-mid log phase of growth before or after a cold-shift as indicated by the experiment. Cells (1 ml) were fixed at room temperature for 1-2 hr with 3.7% formaldehyde added directly to the growth media. Cells were pelleted briefly in a microcentrifuge and then washed 3 times in 0.5ml sorbitol-phosphate buffer (1.2 M sorbitol, 0.1M potassium phosphate, pH 7.5). Cells were resuspended in 100 ul of the same solution containing 40 mg/ml zymolyase (100T) and 0.2% (v/v) b-mercaptoethanol and incubated at 37° for approximately 40 min to digest the cell wall (digestion time varied with strain). Cells were post-fixed for 2′ by adding an equal volume of freshly prepared 8% paraformaldehyde in PBS. 50 ul aliquots of cells were transferred to 16 well NUNC chamber slides that had been pre-coated with a 1mg/ml solution of poly-L-lysine. Excess cell suspension was aspirated off gently and slides were allowed to air-dry slightly. Cells were permeabilized for 6’ with MEOH (-20°) on ice. MEOH was aspirated off and the slide allowed to air dry. Cells were overlaid with 20 ul of Digoxygenin-labeled oligo dT50 probe mix (see Reagents) and incubated at 37° overnight in a humid chamber. The probe mix was aspirated off and immediately replaced with 300 ul 2x SSC. Cells were washed for 20’ at room temperature without agitation. The 2x SSC wash was successively replaced with 1x SSC, 0.5xSSC and antibody blocking solution (1% blocking reagent (w/v) in PBS) for 20′ each. The blocking solution was replaced with 30 ml of mouse monoclonal anti-digoxygenin antibody (1:100 in PBS with blocking agent) and incubated at 37° for 30′. Cells were washed 3 times in 300 ul PBS and incubated for 30′ at 37° with a FITC-conjugated goat anti-mouse antibody (1:400 in PBS). Alternatively, cells were singly stained with a fluorescein-conjugated sheep Fab′ fragments specific for digoxygenin (1:20-1:50 in PBS with blocking agent). Cells were again washed 3 times in PBS prior to mounting in glycerol-gelatin containing DAPI (0.2-0.5 mg/ml) to localize nuclear DNA and p-phenylene diamine (1.25 mg/ml) to retard bleaching.

### Poly A tail length assay

Total RNA was isolated with hot-phenol from cells grown at 30° or shifted to 16° for 3 hr using standard methods. Poly A+ RNA was selected from 150 mg of total RNA by a batch method using Oligotex resin according to manufacturer’s instructions. Poly A+ RNA samples were resuspended in 5 ul of labeling mix and 3′ end-labeled with pcp according to standard methods; the labeling reaction was stopped with the addition of 50ul NaCl soln (500 mM NaCl, 10 mM EDTA). 25 ul of each labeling reaction was digested with a mixture of RNAse A (50 mg/ml) and T1 (1unit/ml) to cleave at all nucleotides except adenine. The resulting poly A tails were run on a 12% polyacrylamide, 7 M urea gel and exposed to Hyperfilm-mp autoradiographic film.

### Cloning and sequence analysis of BRR7/NUP188

A complementing *BRR7* clone was obtained by transforming the outcrossed *brr7-1* mutant with DNA from the Rose genomic library consisting of wild type yeast genomic DNA carried in the *URA3*-marked centromeric vector YCp50 (Rose *et al.*, 1987). The transformation reaction was plated directly on pre-chilled plates lacking uracil and incubated at 16° for 6 days. Failure of transformants to grow on 5-FOA media confirmed that rescue was plasmid-dependent. The original complimenting clone was digested with ClaI and religated to yield an approximately 10 Kb insert in YCp50 (pDBK10) that fully complemented the cs growth and mRNA export defects.

Short stretches of sequence data obtained from the pDBK10 clone were compared with the yeast genome database and an exact match was identified on chromosome XIII in a region containing the *NUP188* ORF (YML103C). Generation of additional subclones identified a minimal complementing fragment (HindIII-ClaI, approximately 6.3 Kb) containing the complete *NUP188* ORF and approximately 300 bp at the 5′ end of the 3.383 kb *MDMI* ORF (pDBK11). Linkage between the *brr7-1* mutation and *NUP188* was confirmed by integration of a wild type copy of *NUP188* along with the *URA3* marker at the *NUP188* locus in the yDBK5 (*brr7-1*) haploid using the pDBK17 integrating plasmid. A stable Ura+ integrant was isolated (yDBK17). All spores from 40 tetrads obtained from a subsequent cross with YPH399 showed wild-type growth at 16°. The failure to recover the cs phenotype among the spores indicated linkage between the mutation and the *NUP188* locus.

### Disruption of the NUP188 gene

The ClaI-Eag1 fragment from pDBK10 containing the *NUP188* ORF and flanking YCp50 sequence was subcloned into bluescript to give the pDBK18 clone. A 1 kb Hpa1 fragment spanning the start of the NUP188 coding sequence was replaced with a linker containing a BamHI site. A subsequent BamHI, XbaI digest allowed replacement of all but approximately the last 200 bp of the *NUP188* ORF with a BamHI-XbaI fragment containing the *LEU2* gene under its own promoter, generating the ∆*nup188* deletion clone pDBK19. The deletion clone was used to PCR a DNA fragment containing the *LEU2* marker along with *NUP188* flanking sequence. The fragment was transformed into the wild type diploid strain, YPH399, in a standard one-step gene replacement procedure. Diploids containing the disruption were selected on media lacking leucine. The disruption was confirmed by Southern analysis. Following sporulation of the heterozygous diploid, the progeny from 10 4-spore tetrads showed 2:2 segregation of the *LEU2* marker and 4:0 growth on YEPD at 30°. A Leu+ spore was outcrossed with the wildtype YPH399 strain and the diploids were sporulated. The *LEU2* marker again showed 2:2 segregation. Leu+ sister spores of the a and α mating types were isolated and designated *nup188∆*::*LEUa* and α.

### In vivo GFP localization

Saturated cultures of cells containing SV40 NLS-GFP, Nab2-GFP, L25 NLS-GFP, or Npl3-GFP constructs were grown in appropriate selective media at RT or 30° depending on the yeast strain. Cells were diluted (1:10-1:50) in selective media and grown to early log phase. Cells were concentrated by brief microcentrifugation prior to live fluorescence microscopy.

### Immunofluorescence localization of Srp1

Cells were grown as for the live localization studies and fixed as in the *in situ* hybridization experiments above. Staining of fixed cells was carried out using an anti-Srp1 rabbit polyclonal antisera (1:100) followed by a TRITC-conjugated goat anti-rabbit antibody (1:1000).

### Fluorescence Microscopy

Fixed and live cells were examined using a Zeiss Axioskop microscope equipped with a 100X (1.3 NA) plan-neofluar oil objective and a 100W mercury lamp. Images documenting the mRNA export defects were photographed using Kodak TMAX 400 film. All samples in a data set were photographed and processed using the same parameters. Film was developed using Edwal developer and digitized using a 35mM negative scanner (Polaroid). Data from protein localization experiments were obtained as 12-bit black and white images collected using a Sensys CCD camera (Photometrics) and displayed with IP Lab software (Scanalytics). All images from a given data set were scaled identically and converted to 8-bit images. Figures were prepared using Adobe Photoshop and Illustrator software.

### Electron Microscopy

Electron microscopy was carried out on YPH399 and *nup188-brr7* cells grown to early log phase at 30° in YEPD and shifted to 16° for 2.5 hr. Cells (20mls, OD_600_≅0.4) were fixed 5′ by adding formaldehyde (4.8%) at incubation temperature before harvesting by centrifugation. Cells were resuspended in cold fixation solution (2% glutaraldehyde, 2%formaldehyde in PM buffer (40mM potassium phosphate (pH 6.5), 0.5mM MgCl_2_)) and incubated for 30’ on ice. Cells were rinsed in sorbitol-phosphate buffer and incubated in the same buffer containing 80mg/ml zymolyase (100T) and 0.2% (v/v) b-mercaptoethanol for 30’ at 37°. Cells were washed 2x in cacodylate buffer (0.1M cacodylate, pH 6.5, 1mM MgCl_2_, 5mM CaCl_2_) and incubated in fresh 0.5% OSO_4,_ 0.8% potassium ferricyanide on ice 5′. Cells were pelleted, resuspended in same, and incubated on ice (total incubation = 15’). Cells were washed 3x5′ in cold dH_2_O and dehydrated with a 30–100% ETOH series at -20° followed by warming to RT and further incubation in 100% ETOH (5′) and 1:1 ETOH:Spurr’s resin (30’) prior to baking 16hr in Spurr’s resin. Acid fast green was added to initial ETOH step to aid visualization of cells. Thin sections of samples were stained en grid first with 1% uranyl acetate (25’) followed by a dH2O wash (37°) and then with lead citrate(2x4’) followed by 3 NaOH (0.01%) washes. Samples were imaged on a Tecnai20 electron microscopy.

### Reagents

Digoxygenin-11-uridine-5′-triphosphate (Dig-11-dUTP), Digoxygenin-specific mouse antibodies and antibody blocking reagent (Boehringer Mannheim), fluorescein (FITC)-conjugated goat anti-mouse antibodies (Cappel, Organon Teknica), zymolyase (ICN), terminal transferase (USB), RNAse A (Sigma), RNAse T1 (Boehringer Mannheim), Oligotex (Qiagen), formaldehyde (Fluka), paraformaldehyde (Sigma), EM grade glutaraldehyde (VWR), Spurr’s resin (Sigma Aldrich), glycerol-gelatin (Sigma), b-mercaptoethanol (Sigma), vanadyl ribonucleoside complex (Gibco BRL), p-phenylenedamine (Sigma), 4,6-diamidino-2-phenylindole (DAPI, Sigma), poly-L-lysine (Sigma), 16 well NUNC chamber slides (American Scientific), Hyperfilm-mp (Amersham).

dT50 Probe preparation: oligo dT50mers were end labeled with Dig-11-dUTP using terminal transferase as described in the product information. 40 ul of Digoxygenin-labeled dT50 probe (approximately 0.5 pmoles/ml) was added to 500 ul 2x hybridization mix (10x SSC, 1% Denhardts, 0.02% tween, 20 mM vanadyl complex in DEPC-treated gdH2O), 140 ul DEPC-treated gdH2O and 300 ul formamide to yield the working probe mix. Negative control probes consisting of random oligo 50mers were end labeled with digoxygenin in a similar manner and used to verify probe specificity in the *in situ* hybridization assay.

### Data availability

For Guthrie lab strain and plasmid requests, please contact Professor Hiten Madhani, Biochemistry and Biophysics Department, UCSF (hitenmadhani@gmail.com). The authors affirm that all data necessary for confirming the conclusions of the article are present within the article figures and tables.

## Results

### Identification of seven candidate cold-sensitive mRNA export mutants

A screen for cs transport mutants was carried out using an existing bank of cs mutants (Moir *et al.* 1982). Cells were grown to early log phase at 30° and then shifted to 16° for 10 hr. Shifted and unshifted cells were assayed for accumulation of bulk poly A RNA by *in situ* hybridization with a digoxygenin-labeled oligo dT50 probe followed by indirect immunofluorescence with an antibody against digoxygenin. Candidate transport mutants showed distinct nuclear staining at 16°. In contrast, wild type cells showed whole-cell or cytoplasmic staining at both temperatures; thus, nuclear mRNA accumulation was not a general effect of incubation in the cold but rather a defect specific to the cs mutants. Seven strains were identified that showed an mRNA export defect after the 10 hr shift. Crosses among the cs mutants identified 6 complementation groups. All diploids were viable at 16°, indicating recessive mutations in all cases.

Three of the candidate transport mutants were also identified in a splicing screen carried out using the same cs bank of mutants (*brr3-1*, *brr4-1* and *brr8-1*, Noble and Guthrie 1996; S. Noble and C. Guthrie, unpublished data). Given the overlap between the two screens, we chose to continue the BRR (Bad Response to Refrigeration) gene nomenclature used in that screen. The candidate transport mutants (*brr3-1*, *brr4-1*, *brr6-1*, *brr6-2*, *brr7-1*, *brr8-1*, *brr9-1*) were backcrossed against the bank parent (DBY473 or DBY460) derived from the S288C strain, and then outcrossed at least once against the wild-type strain YPH399, also derived from S288C. Ten tetrads from each final cross were assayed for growth and nuclear mRNA accumulation at 16°. The transport defects co-segregated with the cs growth defects in all of the mutants. Each tetrad contained two mutant and two wild-type spores, indicating that the defects were caused by single genes.

### How rapid are the mRNA export defects in the cs mutants?

**Figure 1:**
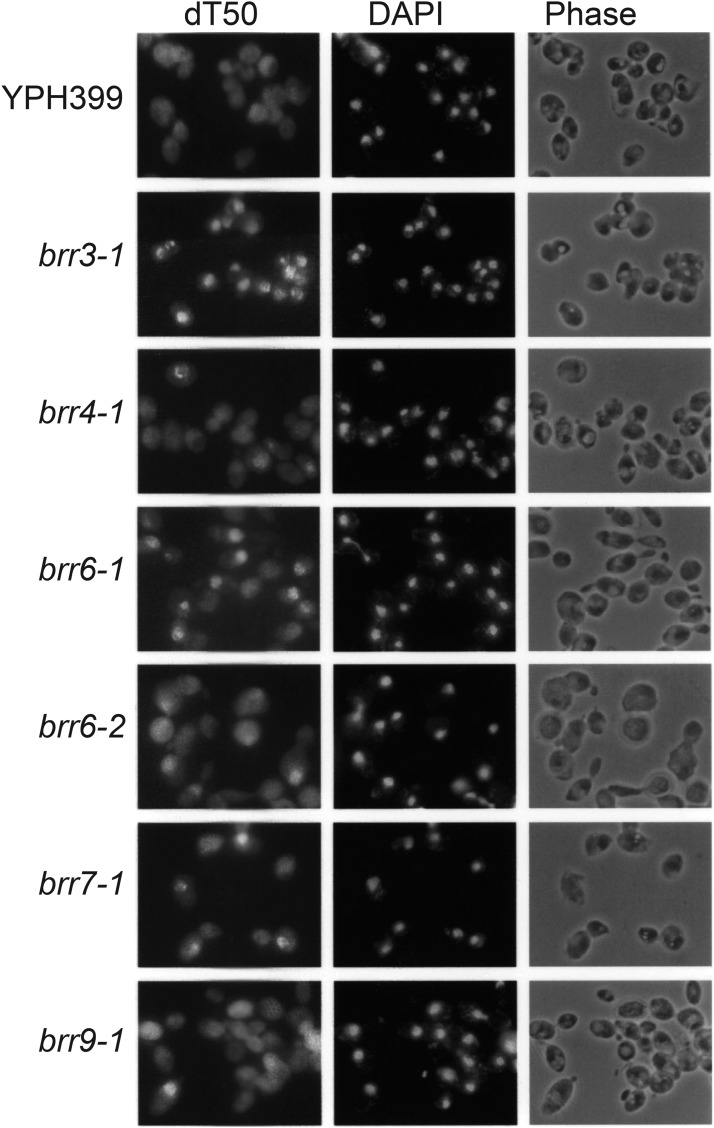
mRNA Localization in candidate cs mRNA export mutants. Shown are the mRNA localization patterns in the cs mutants incubated for 2 hr at 16° C and assayed by *in situ* hybridization with a digoxygenin-labeled oligo dT50 probe. Nuclear location was confirmed by DAPI staining to identify DNA.

A number of ts mutants have previously been found to accumulate mRNA in the nucleus following a temperature shift. Export defects appear within different time frames in different mutants. Some strains, such as the karyopherin mutant *xpo1-1* (Stade *et al.* 1997), accumulate nuclear RNA in minutes while others, such as *nup49-313* (Doye *et al.* 1994), take as much as 5 hr to show a defect. In part, this range may reflect differences in the roles individual proteins play in transport; for example, components directly involved in transport events might be predicted to give rise to the most rapid defects. To further characterize the cs mutants, we carried out detailed hybridization time-courses. Cells were grown to mid-log phase at 30° and shifted to 16° for 0’, 15’, 30’, 1h, 2 hr, 4h, and 6h prior to *in situ* hybridization. Six strains showed detectable nuclear mRNA accumulation by 2 hr (Figure 1); *brr3-1* and *brr7-1* also showed export defects by 15’ and *brr6-1* by 1h (Table 2). The *brr9-1* mutant showed accumulation in a small percentage of cells at 2 hr with increasing penetrance at later times while the *brr8-1* mutant (not shown) did not accumulate mRNA in the nucleus until 4 hr following a cold-shift (Table 2).

**Table 2:** Phenotypes of cold-sensitive mutants isolated in this study

cs Mutant	export defect onset	Splicing Defect*	Tail Length Defect
*brr3-1*	≤ 15 min.	Strong	Strong
*brr4-1*	1-2 hr.	Weak	None
*brr6-1*	1-2 hr.	None	None
*brr6-2*	1-2 hr.	None	None
*brr7-1*	≤ 15 min.	None	None
*brr8-1*	≥ 4 hr.	Weak	None
*brr9-1*	2-4 hr.	none	None

* Awabdy 1997; Noble and Guthrie 1996; S. Noble and A. de Bruyn Kops, unpublished data.

### Do the mutants show defects in other aspects of mRNA metabolism?

Messenger RNA export is highly coupled with various types of processing occurring during mRNA maturation, including splicing and polyadenylation (reviewed, Katahira 2015). Therefore, we asked if these processes were also affected in the mutants. Approximately 5% of yeast transcripts, including the abundant mRNAs encoding ribosomal proteins, undergo splicing. In the earlier screen for cold-sensitive splicing mutants carried out in our lab, *brr3-1* showed strong splicing defects while *brr4-1* and *brr8-1* strains exhibited weak effects following a 10 hr shift to 16° ((Noble and Guthrie 1996); S. Noble and C. Guthrie, unpublished data). In subsequent experiments, *brr3-1* also showed a strong splicing defect when cells were shifted for 15’ and 3 hr while *brr6*, *brr7*, *8*, and *9* showed no effect ((Awabdy 1997) and A. de Bruyn Kops, unpublished data). The splicing effects are summarized in Table 2.

**Figure 2:**
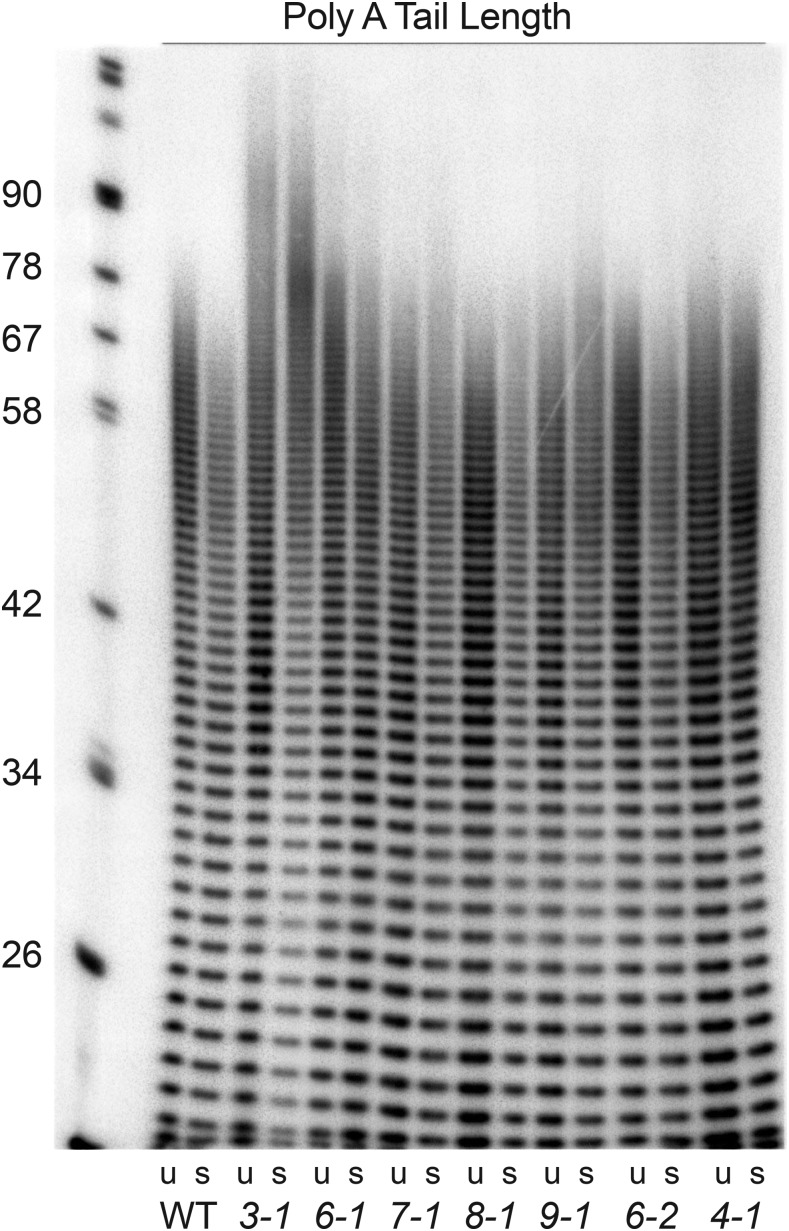
Poly A tail length in candidate cs mRNA export mutants. Shown are the results of poly A tail length assays carried out using RNA from cells grown at 30° C (u) or incubated at 16° C for 3 hr (s). The *brr3-1* strain shows a population of longer poly A tails (approximately 80-90 nt) not seen in RNA samples from wild-type cells or the other cs mutant.

Regardless of whether or not they undergo splicing, all yeast mRNAs acquire a poly A tail of approximately 80-90 nucleotides in a processing step that is coupled to termination and release of the nascent mRNA from the site of transcription (Sachs and Davis 1989; Minvielle-Sebastia *et al.* 1997). In wild type cells, poly A tails are rapidly deadenylated to about 70 nts while longer tails (80-90 nts) were shown to persist in a number of candidate ts transport mutants (Kadowaki *et al.* 1994). To determine if poly A tail length was affected in the cs mutants, poly A+ RNA was assayed by 3′ end labeling followed by RNAse A and T1 digestion. Tails of approximately 80-90 nt were observed in *brr3-1* cells at both 30° at 16° (Figure 2). The proportion of longer tails in *brr3-1* increased significantly following the cold shift. Long tails were not observed in wild type cells or in any of the other cs mutants, indicating that increased tail length is not a general effect of the cold shift. Given that *brr3-1* is an allele of the key mRNA export gene *GLE1* (see below) and shows a rapid and severe export defect (Figure 1 and Table 2), the presence of robust splicing and tail-length defects in *brr3-1* may reflect decreased expression of essential RNA processing factors owing to impaired mRNA export. The absence of splicing and poly A tail length in *brr7-1*, where an early and strong export defect is also detected, could point to effects on different populations of mRNA.

### Cloning of BRR3, BRR6, and BRR7

Cloning of the genes for the *brr3*, *brr6* and *brr7* mutants was carried out by complementation of the cs growth defects using a genomic DNA library (Rose *et al.* 1987). The *brr3* mutant was shown to be a novel allele of *GLE1* (Awabdy 1997), a key export factor that functions in the release of exported mRNAs from the cytoplasmic face of the NPC (Del Priore *et al.* 1996; Murphy and Wente 1996; York *et al.* 1999; Kendirgi *et al.* 2003; Lund and Guthrie 2005; Alcázar-Román *et al.* 2006; Weirich *et al.* 2006; Tran *et al.* 2007, reviewed, Folkmann *et al.* 2014). The *brr6-1* and *brr6-2* strains proved to carry identical mutations of the *BRR6* gene that we showed encodes a nuclear envelope transmembrane protein (de Bruyn Kops and Guthrie 2001). Brr6 has been further characterized elsewhere (Scarcelli *et al.* 2007; Hodge *et al.* 2010; Tamm *et al.* 2011; Lone *et al.* 2015; Zhang *et al.* 2018, reviewed, Schneiter and Cole 2010; Jaspersen and Ghosh 2012) and we have recently found that the mRNA export defect in *brr6-1* stems from impaired transcriptional regulation of the major poly A binding protein gene, *PAB1*, that is required for export (A. de Bruyn Kops, J. E. Burke and C. Guthrie, unpublished data). The complementing clone for *brr7-1* (see Materials and Methods) corresponded to the previously identified *NUP188* coding sequence (Nehrbass *et al.* 1996; Zabel *et al.* 1996). Henceforth, we refer to this cs mutant as *nup188-brr7*.

### The nup188-brr7 mutant shows selective defects in mRNA export and protein import

**Figure 3:**
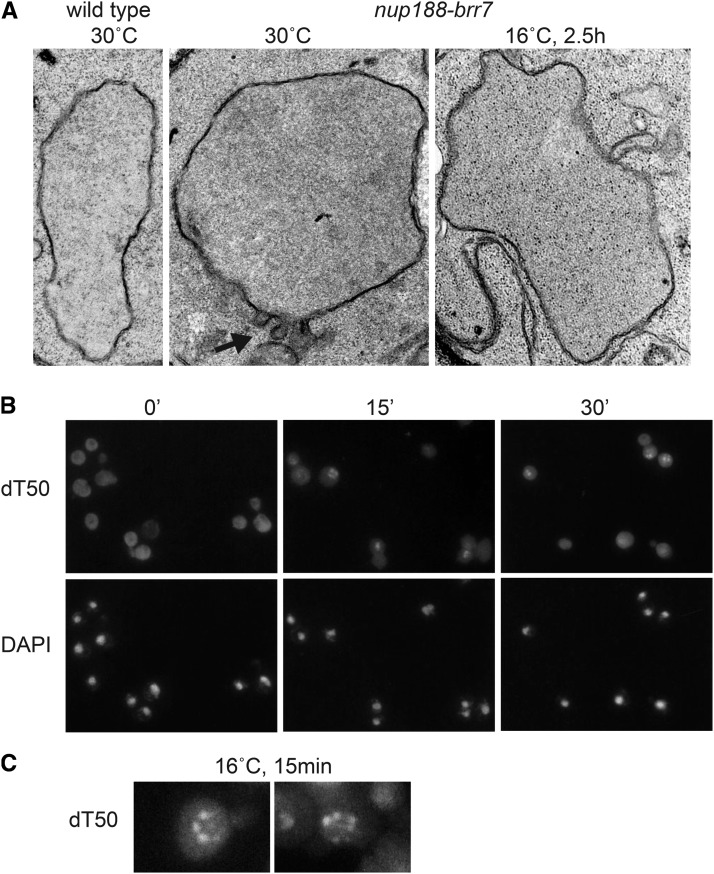
mRNA localization phenotype in *nup188-brr7*. Panel A: shows electron microscopy images of wild type and *nup188-brr7 1* nuclei. Black arrow indicates nuclear envelope abnormalities. Panel B: shows mRNA accumulation detected by dT50 *in situ* hybridization in *nup188-brr7* at early times (0 min- 30’) following a shift to 16° C. DAPI staining shows the location of the cell nuclei. Panel C: shows two enlarged examples of the nuclear rim-staining pattern observed in *nup188-brr7* at early times. At later times (2-6 hr), the mRNA staining becomes nucleoplasmic (Figure 1).

The Nup188 protein has been localized to both faces of the nuclear pore in isolated nuclear membrane preparations using immuno-electron microscopy (Nehrbass *et al.* 1996). A role for Nup188 in mRNA transport was unanticipated given that previous studies on *nup188* ts (*psl4*) and deletion (*nup188∆*::*HIS3*) alleles failed to detect mRNA export defects. Instead, The *nup188-psl4* allele (but not ∆*nup188*::*HIS3*) was shown to have dramatic defects on nuclear envelope morphology, leading to the conclusion that the Nup188 protein plays a role in nuclear pore structure but not in transport *per se* (Nehrbass *et al.* 1996). In contrast, the *nup188-brr7* allele showed mild effects on membrane morphology at 30° that were not substantially increased after 2.5 h at 16° (Figure 3A).

**Figure 4:**
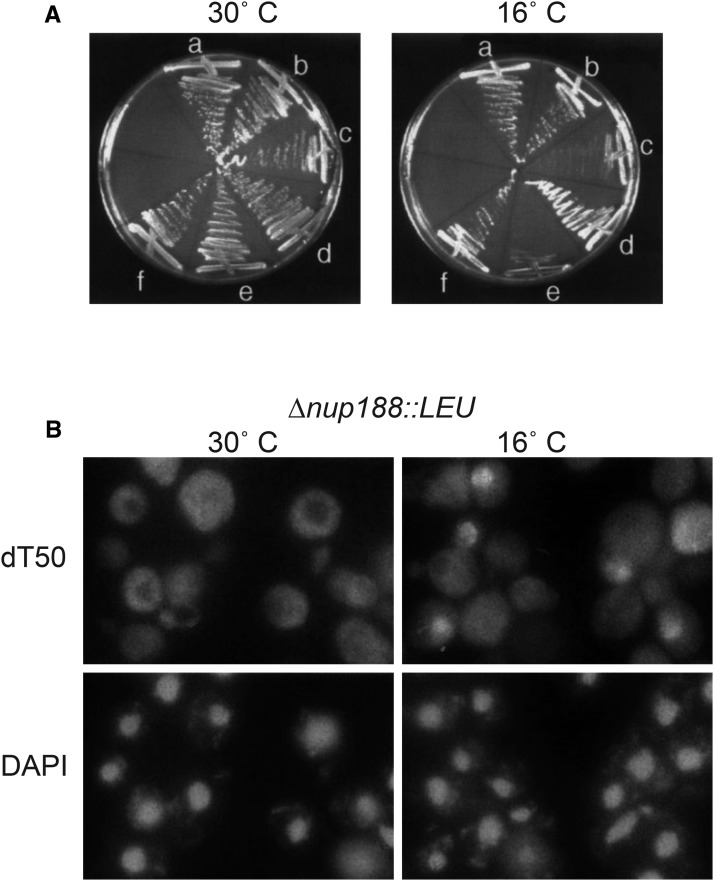
Growth and mRNA localization phenotypes in *nup188*∆*::LEU2*. Panel A: shows the growth phenotypes of YPH399 wild type (a,b), *nup188-brr7* (c,d) and *nup188∆*::*LEU2* (e,f) strains at 30° and 16° C. The strains carried either vector alone (a,c,e) or a wild-type NUP188 plasmid (b,d,f). Panel B: shows the nuclear mRNA accumulation phenotype observed in the *nup188∆*::*LEU2* deletion strain assayed by dT50 *in situ* hybridization following a 2 hr incubation at 16° C. The mRNA export defect was observed in approximately 30–70% of the cells in different experiments. DAPI staining shows the location of the cell nuclei.

Like the *psL4* allele, *nup188-brr7*showed no export defect at 30°; however, clear nuclear accumulation of mRNA was detected in ≥20% of cells at 16° by 15’ (Figure 3B) and ≥70% of cells by 2 hr (Figure 1). The similarity in *nup188-brr7* envelope morphology at the two temperatures argues against the membrane effects causing the export defect. Notably, the nuclear mRNA that accumulated in *nup188-brr7* was concentrated in foci at the nuclear periphery at early times (Figure 3C). At later times after a cold-shift (3-6 hr), nucleoplasmic staining predominated (data not shown), suggestive of an initial accumulation of mRNA at the nuclear pores followed by a backup in the nucleoplasm as more mRNA accumulated. Interestingly, a gene disruption in the *NUP188* locus in the YPH399 strain background (∆*nup188*::*LEU2*) also exhibited cs growth (Figure 4A) and export defects (Figure 4B). The effects of the *nup188-brr7* and ∆*nup188*::*LEU* cs mutants suggest a connection between Nup188 and mRNA export not revealed by previous ts alleles.

Like mRNA export, import of proteins into the nucleus occurs through the NPC, yet these processes are distinct. Whereas the majority of mRNA export occurs via the well-defined Mex67/TAP pathway (reviewed, Katahira 2015), specific carrier proteins (karyopherins) that recognize various protein nuclear localization signals (NLS) interact with the NPC to mediate import of different classes of proteins (reviewed, Floch *et al.* 2014). To ask whether the *nup188-brr7* and *nup188∆*::*LEU2* mutants affect protein import as well as mRNA export, we examined the localization of transport substrates for 4 different protein import pathways mediated by: 1) Kapβ(Kap95)/Kapα(Srp1) (“classical” NLS-protein”) 2) Mtr10 (*e.g.*, Npl3), 3) Kap104 (hnRNP proteins, *e.g.*, Nab2), and 4) Kap123 and Kap121/Pse1 (ribosomal protein, *e.g.*, L25).

**Figure 5:**
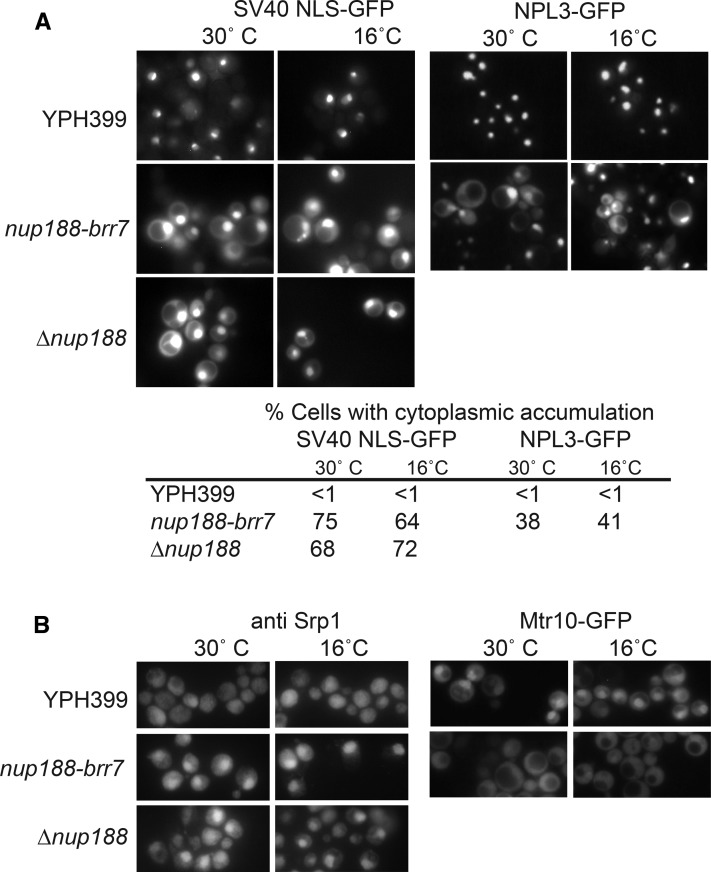
SV40 NLS-GFP and Npl3-GFP Localization in *nup188-brr7* and *nup188∆*::*LEU2*. Panel A: shows GFP localization in live *nup188-brr7* and *nup188∆*::*LEU2* cells containing low copy SV40 NLS-GFP (left) and Npl3-GFP with the Npl3 promoter (right) constructs. Cells were grown at 30° C in selective media and incubated at 16° C for 2 hr. Quantitation of cells showing cytoplasmic accumulation based on scoring ≥100 cells per condition are shown below. Panel B: Shows immunofluorescence with an anti-Srp1 antibody in fixed cells (left) and GFP localization in live cells containing a low copy plasmid carrying Mtr10-GFP (right) under the same conditions.

To assay the “classical” NLS import pathway, we localized an SV40 NLS-GFP fusion protein in living cells. Cells were imaged at exposures that allowed detection of cytoplasmic signal in the linear range, causing the brighter nuclear signal to saturate in many cases; hence we limit our comparisons to the relative cytoplasmic signal in mutant *vs.* wild type cells. Both the *nup188-brr7* and *nup188∆*::*LEU2* mutants showed a steady-state GFP accumulation in the cytoplasm relative to wild-type cells at both 30° and 16° (Figure 5A). This effect was similar to that observed with karyopherin α mutants (Hahn *et al.* 2008). To determine whether the Mtr10-mediated import pathway was also affected in *nup188-brr7*, we localized an Npl3-GFP fusion protein. As with the classical NLS pathway, the *nup188-brr7* mutant showed increased accumulation of Npl3-GFP in the cytoplasm relative to wild type cells. Localization of the reporter was not assayed in the *nup188∆*::*LEU2* strain because of marker incompatibility. The cytoplasmic accumulation in the *nup188* alleles was similar to the Npl3 and Npl3-GFP mislocalization patterns reported for *mtr10* mutants (Pemberton *et al.* 1997; Senger *et al.* 1997).

The percentages of cells showing cytoplasmic accumulation of both reporters based on scoring of ≥100 cells/condition are listed below the images. For both pathways, a striking increase in the proportion of cells with pronounced cytoplasmic signal was observed in the *nup188* mutants, consistent with a block to these import pathways. Notably, the nuclear SV40 NLS-GFP signal also appeared elevated in the mutant, raising the possibility that the increased cytoplasmic signal reflected increased expression rather than an import block. We think it likely that the mutants are, in fact, defective in these import pathways because the carriers for both pathways (Srp1(Kapα) and Mtr10) also show pronounced mislocalization compared with wild type (Figure 5B). Importantly, the mutants resulted in decreased nuclear localization of Mtr10-GFP but increased nuclear Srp1. These results suggest that failed re-export of the Srp1 may limit cytoplasmic availability, causing the SV40 NLS-GFP import defect. Interestingly, Srp1 is also responsible for import of the proteasome (Chen and Madura 2014) required for nuclear protein degradation, possibly explaining increased nuclear SV40 NLS-GFP signal in the mutants.

**Figure 6:**
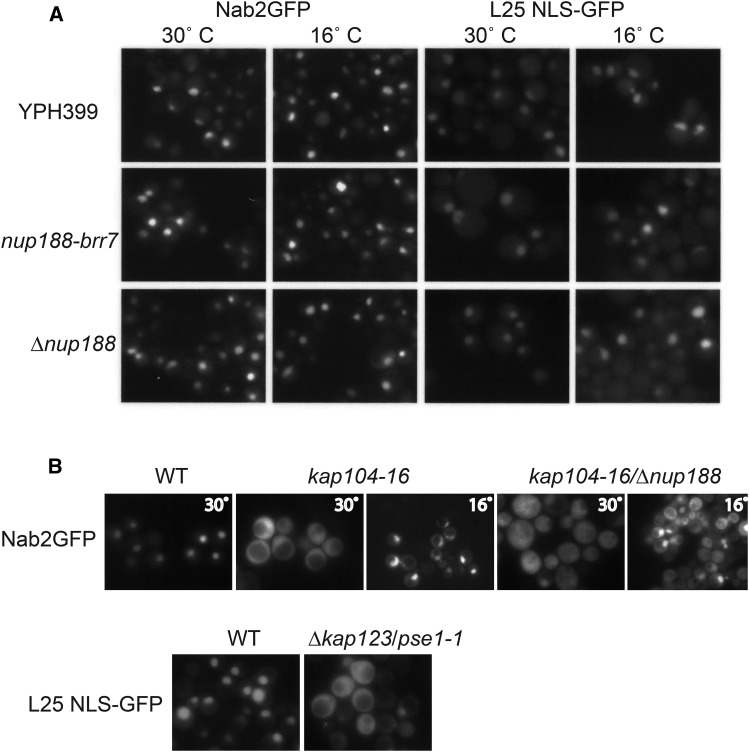
Nab2-GFP and L25 NLS-GFP Localization. Panel A: shows Nab2-GFP (left) and L25 NLS-GFP (right) fusion protein localization in separate live experiments using YPH399, *nup188-brr7* and *nup188∆*::*LEU2* strains containing low copy constructs. Cells were grown at 30° C and then shifted to 16°C for 2 hr. Panel B: shows control experiments in which Nab2-GFP (top) and L25 NLS-GFP (bottom) were localized in *kap104-16* and ∆*kap123/pse1-1* strains respectively. Cells were grown at room temperature (RT) and imaged after a shift to 37° C for 30’ (∆*kap123/pse1-1* mutant, and isogenic wild type) or 30° for 1 hr (*kap104-16*, ∆*nup188*::*LEU2/kap104-16* double mutant, and wild type isogenic to *kap104-16*) or 16°C for 2h (*kap104-16* and ∆*nup188*::*LEU2/kap104-16* double mutant). Both reporters show striking cytoplasmic accumulation in these controls.

To ask if other import pathways are affected in the *nup188* mutants, we used Nab2-GFP and L25 NLS-GFP reporters to test for defects in the Kap104 and Kap123/Pse1(Kap121) pathways respectively. The *nup188* alleles showed normal localization of both reporters at 30° and 16°, in >90% of cells (Figure 6a). In contrast, striking import defects were observed in the control strains ∆*kap123*/*pse1-1-ts* at 37° and *kap104-ts* and a ∆*nup188/kap104-ts* double mutant at 30° (Figure 6b), confirming that defects can be detected with these reporters. Surprisingly, although the *kap104-ts* mutant on its own showed nuclear accumulation of the Nab2 reporter at 16° the double mutant showed a mixed phenotype. We speculate the absence of Nup188 may sensitize the cells to the *kap104-16* mutation. These results show that the Kap104 and Kap123/Pse1(Kap121) import pathways are unaffected in the *nup188* alleles. The mislocalization of mRNA and the SV40 NLS-GFP and Npl3-GFP reporters but not Nab2-GFP or L25 NLS-GFP suggest that Nup188 has selective effects on different transport pathways.

## Discussion

Using a dT50 *in situ* hybridization assay to screen a bank of cs yeast strains for mutants that accumulated bulk poly A RNA in the nucleus, we identified seven candidate cs transport mutants in six complementation groups (*brr3*, *brr4*, *brr6*, *brr7*, *brr8* and *brr9*) that showed mRNA export defects following a cold shift. With the exception of *brr3-1*, these mutants showed little or no effect on splicing or poly A tail-length. Thus, their roles in transport are likely to be independent of these other steps in the mRNA maturation pathway.

We identified complementing genes for 3 of the mutants (*brr3-1*, *brr6-1* and *brr7-1*): *brr3-1* (Awabdy 1997) is a novel cs allele of the export factor *GLE1* that functions in release of mRNA cargoes on the cytoplasmic face of the NPC (Del Priore *et al.* 1996; Murphy and Wente 1996; York *et al.* 1999; Kendirgi *et al.* 2003; Lund and Guthrie 2005; Alcázar-Román *et al.* 2006; Weirich *et al.* 2006; Tran *et al.* 2007, reviewed, Folkmann *et al.* 2014). The *BRR6* gene encodes a nuclear envelope transmembrane protein (de Bruyn Kops and Guthrie 2001; Scarcelli *et al.* 2007; Hodge *et al.* 2010; Tamm *et al.* 2011; Lone *et al.* 2015; Zhang *et al.* 2018, reviewed, Schneiter and Cole 2010; Jaspersen and Ghosh 2012); we recently found that the export defect in this strain stems from impaired transcriptional regulation of the major poly A binding protein gene, *PAB1* (A. de Bruyn Kops, J. E. Burke and C. Guthrie, unpublished data). *brr7-1*(*nup188-brr7*) is a novel cs allele of the nucleoporin, *NUP188* (Nehrbass *et al.* 1996; Zabel *et al.* 1996).

### nup188-brr7 accumulates mRNA at the nuclear rim and impairs select protein import pathways

The onset of the mRNA export defect in *nup188-brr7* (Figure 3) occurs rapidly after a cold-shift, consistent with a close connection to transport mechanisms. The accumulation of mRNA at the nuclear rim at early times could reflect mRNA association with the NPC. The rim-staining pattern was also observed in the *nup188*∆*::LEU2* strain (data not shown), implying that Nup188 is not necessary for this association. In addition to the cs mRNA export phenotype, *nup188-brr7* and *nup188∆*::*LEU2* also showed significant defects in nuclear the Kap95/Srp1 (Kap α/β) and Mtr10(Kap111) but not Kap104 and Kap123/Pse1(Kap121)-mediated import pathways at both 30° and 16°. We observed cytoplasmic accumulation of the NLS-GFP and Npl3-GFP reporters that utilize these pathways as well as mislocalization of the carriers themselves. Interestingly, the mislocalization of the carriers revealed that, although Mtr10 import was blocked as expected, Srp1(Kapα) aberrantly accumulated in the nucleus. This suggests that the SV40-NLS-GFP import defect may stem from failure to recycle Srp1, rather than from a block to Kap α/β-mediated import *per se*. Since the *nup188∆*::*LEU2* deletion strain showed cs growth and transport defects similar to those observed in the *nup188-brr7* mutant, these phenotypes most likely reflect the loss of Nup188 function.

Nup188 and its paralogue, Nup192 are components of the yeast Nic96 complex (reviewed Vollmer and Antonin 2014) that comprises part of the central scaffold of the NPC. This complex also contains two other pairs of proteins regarded as paralogues, Nup53 /Nup59, and Nup157/170 (Nehrbass *et al.* 1996; Marelli *et al.* 1998; Theerthagiri *et al.* 2010; Amlacher *et al.* 2011), and is conserved in higher eukaryotes (Nup93, Nup188/Nup205, Nup53/Nup35, Nup155; reviewed, Vollmer and Antonin 2014). The Nic96 complex interacts with the nuclear envelope and also with FG repeat-containing proteins comprising a hydrogel mesh in the center of the pore. In particular, nic96 was shown to anchor the Nsp1-Nup57-Nup49 FG repeat containing protein complex (Schlaich *et al.* 1997; Schrader *et al.* 2008). In addition, a recent study showed that the GLFG subclass of FG Nups binds to Nup192 and Nup188, affecting NPC assembly and possibly stability (Onischenko *et al.* 2017).

FG repeat proteins are known to interact with karyopherins as well as with the major mRNA export carrier, Mex67 (reviewed, Wälde and Kehlenbach 2010; Adams and Wente 2013; Floch *et al.* 2014). Studies using collections of ∆FG repeat nucleoporin mutants (Strawn *et al.* 2004; Terry and Wente 2007) established that different combinations of specific FG repeat proteins are required for different transport pathways. Notably, ∆FG mutants that affected either the Kap104 or the Kap121 pathways had no effect on the Kap α/β import, mRNA export or Cse1-mediated NES export pathways. In light of these distinctions, it is interesting that we observed effects on mRNA export but not Kap104 and Kap121 import pathways.

Various results connect the Nic96 complex with both transport and molecular sieve functions of the pore. Deletion of *NUP188* and *NUP170* increases NPC permeability in yeast (Shulga *et al.* 2000; Shulga and Goldfarb 2003) and similar effects have been seen with loss of the homologous Nup93 complex in metazoans (reviewed, Vollmer and Antonin 2014). In addition, Nup53 plays a cell cycle-dependent role in the Kap121 protein import in yeast (Marelli *et al.* 1998; Makhnevych *et al.* 2003; Cairo *et al.* 2013) and down-regulation of Nup93 and Nup205 (homologs of yeast Nic96 and Nup192) impairs import of Smad cargoes in Drosophila (Chen and Xu 2010). Finally, repression of *NUP192* and certain *nup192* mutants (see below) impairs import of a Nab2 NLS reporter (Sampathkumar *et al.* 2013) and mRNA export (Stuwe *et al.* 2014) respectively.

These observations, as well as the transport defects in *nup188-brr7*, could reflect a role for the Nic96 complex in recruiting the FG repeat proteins to the NPC. In the ∆FG mutant studies (Strawn *et al.* 2004; Terry and Wente 2007), mRNA export was strongly linked to the FG repeats of the symmetrically located nup57 and nup49 proteins and those of Nup1 and Nup2 located at the inner face of the NPC. Given that the Nic96 complex anchors the complex containing Nup57 and Nup49, it is possible that the *nup188* mutants perturb mRNA export by altering these interactions. Consistent with this idea, a recent study demonstrated plasticity in NPC composition, showing that altered expression of Nic96 complex proteins including Nup188 can alter nucleoporin stoichiometry and structure of the NPC (Rajoo *et al.* 2018).

In addition, direct roles for the Nic96 complex in transport are also possible; in fact, Nup53 has been shown to bind specifically to the karyopherin Kap121 (Marelli *et al.* 1998)and to karyopherin α (Stuwe *et al.* 2014). Recent studies of Nup188 and Nup192 have demonstrated structural similarities with nuclear transport receptors, suggesting an evolutionary relationship between these proteins and karyopherins (Amlacher *et al.* 2011; Flemming *et al.* 2012; Andersen *et al.* 2013). Furthermore, soluble fragments of the Nup188 and Nup192 proteins, like karyopherins, were shown to bind to FG repeats in the pore channel and translocate through the pore (Andersen *et al.* 2013). Although Nup188 and Nup192 are stably associated with the NPC, and therefore presumably not functioning as carriers, it was suggested that interaction with FG repeats could be necessary for correct formation of the permeability barrier.

Interestingly, an NLS-like domain of Nup53 binds to the tail region of Nup192 in an interaction that is mutually exclusive with Nup53-karyopherin α association (Stuwe *et al.* 2014). Mutants of Nup192 that abolished Nup53 binding showed significant growth and mRNA export defects, leading to the suggestion that these interactions may function in regulation of transport. Although the connection between Nup188 and transport is less well explored, Nup188 shares the karyopherin-like structure of Nup192 and also binds Nup53, suggesting that it could impact transport in similar ways.

## Future Directions

We envision that further studies using the *nup188-brr7* mutant may prove useful in dissecting the regulation of transport through the NPC and the role of the Nic96 complex. Our preliminary experiments suggest a number of avenues worth exploring. First, our results suggest a defect in re-export of Srp1, a process that involves the karyopherin Cse1 and Nup2 (Solsbacher *et al.* 1998; Booth *et al.* 1999). Notably, Nup2 shows steady state localization at the nuclear pore face but is the sole mobile nucleoporin in yeast, raising the possibility of other locations (Denning *et al.* 2001; Dilworth *et al.* 2001). Given the potential for *nup188* mutants to alter the Nup composition in the pore, localization of Nup2 and Cse1 in the *nup188* mutants as well as further dissection of requirements for specific nucleoporins in the Cse1 pathway would be valuable.

A second avenue would further explore the mRNA export defect in the *nup188-brr7* mutant. This effect could reflect impaired interactions of the major mRNA export carrier, Mex67, with the NPC as Mex67 is known to bind to Nup57 FG repeats (Terry and Wente 2007). Wild type Mex67 protein shows steady state localization at the nuclear rim while the Mex67-5 mutant protein accumulates in the cytoplasm (Segref *et al.* 1997). Although in early experiments mRNA accumulation in intranuclear foci in *mex67-5* (Segref *et al.* 1997; Santos-Rosa *et al.* 1998; Hurt *et al.* 2000), recent studies including single molecule localization showed that this mutant accumulates mRNA at the envelope and strongly affects the release of mRNA from the cytoplasmic face of the NPC (Smith *et al.* 2015; Paul and Montpetit 2016). Single molecule localization of Mex67 in *nup188-brr7* to characterize the export block would be valuable as failure of Mex67 to either transit the channel, release from the cytoplasmic face or re-enter the nucleus could all lead to the *nup188-brr7* phenotype. Single molecule mRNA localization in *nup188-brr7*, *mex67* double mutants could show if the *nup188-brr7* block precedes that in *mex67-5*, helping to dissect the steps of mRNP transit through the NPC. Along the same lines, exploring mRNA localization in *nup188-brr7 and brr3-1* double mutants might be informative as *brr3-1* is an allele of *GLE1*, a shuttling export factor (Del Priore *et al.* 1996; Murphy and Wente 1996; Kendirgi *et al.* 2003) which functions in dismantling mRNP complexes at the cytoplasmic pore face (York *et al.* 1999; Lund and Guthrie 2005; Alcázar-Román *et al.* 2006; Weirich *et al.* 2006; Tran *et al.* 2007).
